# Association between long interspersed nuclear element-1 methylation levels and relapse in Wilms tumors

**DOI:** 10.1186/s13148-017-0431-6

**Published:** 2017-12-12

**Authors:** Bruna M. de Sá Pereira, Rafaela Montalvão-de-Azevedo, Paulo Antônio Faria, Neimar de Paula Silva, Pedro Nicolau-Neto, Mariana Maschietto, Beatriz de Camargo, Sheila Coelho Soares Lima

**Affiliations:** 1 Post Graduate Program of Instituto Nacional do Cancer (INCA), Rio de Janeiro, Brazil; 2 Pathology Division of Instituto Nacional do Câncer (DIPAT-INCA), Rua Cordeiro da Graça 156, Santo Cristo, Rio de Janeiro, 20220-400 Brazil; 3 Molecular Carcinogenesis Program, Research Center (CPQ), Instituto Nacional do Câncer (INCA), Rua André Cavalcanti 37, Centro, Rio de Janeiro, 20231-050 Brazil; 4 Brazilian Center for Research in Energy and Materials (CNPEM), Brazilian Biosciences National Laboratory (LNBio), Rua Giuseppe Máximo Scolfaro 10.000, Bosque das Palmeiras, Campinas, Sao Paulo 13083-970 Brazil; 5 Pediatric Hematology-Oncology Research Program, Research Center (CPQ), Instituto Nacional de Câncer (INCA), Rua Andre Cavalcanti 37, Centro, Rio de Janeiro, 20231-050 Brazil

**Keywords:** Wilms tumor, LINE-1, Hypomethylation, Relapse

## Abstract

**Background:**

Wilms tumor (WT) is a curable pediatric renal malignancy, but there is a need for new molecular biomarkers to improve relapse risk-directed therapy. Somatic alterations occur at relatively low frequencies whereas epigenetic changes at 11p15 are the most common aberration. We analyzed long interspersed element-1 (LINE-1) methylation levels in the blastemal component of WT and normal kidney samples to explore their prognostic significance.

**Results:**

WT samples presented a hypomethylated pattern at all five CpG sites compared to matched normal kidney samples; therefore, the averaged methylation levels of the five CpG sites were used for further analyses. WT presented a hypomethylation profile (median 65.0%, 47.4–73.2%) compared to normal kidney samples (median 71.8%, 51.5–77.5%; *p* < 0.0001). No significant associations were found between LINE-1 methylation levels and clinical–pathological characteristics. We observed that LINE-1 methylation levels were lower in tumor samples from patients with relapse (median methylation 60.5%) compared to patients without relapse (median methylation 66.5%; *p* = 0.0005), and a receiving operating characteristic curve analysis was applied to verify the ability of LINE-1 methylation levels to discriminate WT samples from these patients. Using a cut-off value of 62.71% for LINE-1 methylation levels, the area under the curve was 0.808, with a sensitivity of 76.5% and a specificity of 83.3%. Having identified differences in LINE-1 methylation between WT samples from patients with and without relapse in this cohort, we evaluated other prognostic factors using a logistic regression model. This analysis showed that in risk stratification, LINE-1 methylation level was an independent variable for relapse risk: the lower the methylation levels, the higher the risk of relapse. The logistic regression model indicated a relapse risk increase of 30% per decreased unit of methylation (odds ratio 1.30; 95% confidence interval 1.07–1.57).

**Conclusion:**

Our results reinforce previous data showing a global hypomethylation profile in WT. LINE-1 methylation levels can be suggested as a marker of relapse after chemotherapy treatment in addition to risk classification, helping to guide new treatment approaches.

**Electronic supplementary material:**

The online version of this article (10.1186/s13148-017-0431-6) contains supplementary material, which is available to authorized users.

## Background

Renal tumors represent 5 to 10% of tumors in childhood, with approximately 93% of these being Wilms tumors (WTs) [1]. In Brazil, the annual incidence rate is approximately 9.4 cases per million [2]. WT is highly curable, with a survival rate of 90% [3], although a subset of patients present with tumor relapse (15–20%); in these cases, overall survival decreases to 50–60% [4]. Currently, two therapeutic approaches are used to treat WT; both present the same survival and relapse rates, differing only in the classification of risk factors. According to The Société Internationale d’Oncologie Pédiatrique (SIOP) protocol, patients receive preoperative chemotherapy whereas in the Children’s Oncology Group protocol, patients undergo surgery as the initial treatment. Risk classification is largely based on tumor stage and histology and is used to guide clinical management [5]. Pre-treated WTs with predominance of the blastemal component classify the patients as high risk [6].

Currently, efforts have been made to identify molecular alterations to be implemented as biomarkers to improve risk stratification. Loss of heterozygosity of both 1p/16q in chemotherapy-naive tumors and gain of 1q in treated and chemotherapy-naive tumors have been associated with an increased risk of relapse/death and were suggested to be incorporated into clinical decisions [7–9].

A remarkable characteristic of WT is its relatively low frequency of somatic mutations, detected in only 30% of the cases, while epigenetic alterations such as loss of imprinting on chromosome 11p15 are observed in 70% of cases [10]. Considering the methylated cytosines as determined by high-performance liquid chromatography, most WTs are hypomethylated compared to adult tissues, although a considerable proportion (49%) have no alteration or moderate hypomethylation, without association with tumor stage [11]. Considering CpG site methylation levels, WTs present a hypomethylation profile compared to matched nephrogenic rests and normal kidneys [12], with specific CpG islands presenting hypermethylation [11, 13]. Genome-wide methylation analyses also have identified three differentially methylated regions (DMRs) capable of correctly distinguishing tumors from normal kidney tissues with a sensitivity of 98% and revealed a significant difference in methylation levels between intermediate and high-risk WT. Given the high prevalence of the DMRs (present in 112/120 WTs examined), these authors presented a pilot study in which DMR-2 could be detected in the circulation of patients with WT, showing potential for clinical utility [14].

The long interspersed element-1 (LINE-1) sequences are retrotransposon elements comprising ∼17% of the human genome, and some of them still retain the capacity to retrotranspose themselves to new genomic locations [15]. LINE-1 is expected to be methylated in normal tissues but presents decreased methylation levels in cancers [16–19], usually related to genomic instability and poor prognosis [16, 20]. In WTs, lower LINE-1 methylation levels have been linked to telomere shortening compared to normal kidneys, without a reported association with clinical data due to the small sample size [21].

In this study, LINE-1 methylation levels were analyzed in WT and kidney samples and explored in the context of identifying current prognostic parameters.

## Methods

### Patients and samples

This study included 47 patients with sporadic, unilateral, and localized WT (without association with congenital anomalies) who were diagnosed and treated according to the SIOP WT 2001 protocol [3] between 2003 and 2014 at the Pediatric Department of Instituto Nacional do Cancer (INCA), Rio de Janeiro, Brazil. All samples were formalin-fixed, embedded in paraffin (FFPE), and stored in the pathology division of INCA. New hematoxylin–eosin-stained slides were reviewed by a pathologist (PAF), who defined viable areas of blastemal component and normal renal cortex tissues. For this study, we selected only the blastemal component of the tumor ignoring other components and overall histological classification for molecular analysis. However, histology was used for risk assessment and tumors were classified according to SIOP guidelines as intermediate or high risk [22]. The presence of anaplasia was not considered because it was very rare (six cases) and not selected as an area for DNA extraction. This study was approved by the Research Ethics Committee of INCA, number 131/13, and informed consent was signed by the children’s guardians.

### DNA extraction and quantification

Two punches (1 mm diameter each) from selected regions were used for DNA extraction using the QIAmp DNA Mini Kit (QIAGEN) according to the manufacturer’s instructions. DNA quantity and purity were assessed by spectrophotometry (Nanodrop).

### LINE-1 methylation analysis

A total of 500 ng of DNA was converted using the EpiTect Plus Bisulfite Conversion kit (QIAGEN) according to the manufacturer’s instructions. Briefly, ~ 50 ng of bisulfite-treated DNA was used as template for a PCR with LINE-1 primers (forward: 5′-biotin-TAGGGAGTGTTAGATAGTGG and LINE-1-reverse 5′-AACTCCCTAACCCCTTAC) and Platinum Taq DNA polymerase (INVITROGEN). Cycling conditions included an initial denaturation at 95 °C for 15 min, followed by 50 cycles consisting of denaturation at 95 °C for 40 s, annealing at 56 °C for 40 s, and extension at 72 °C for 40 s. A final extension step at 72 °C for 10 min was performed. PCR products were then pyrosequenced using the sequencing primer 5′-AACTCCCTAACCCCTTAC in the Pyromark Q96 ID (QIAGEN), following the manufacturer’s instructions. Efficiency of bisulfite conversion was verified using nonCpG cytosine residues as built-in controls.

The pyrosequencing method treats each CpG site as a C/T polymorphism and generates quantitative data (in percentage) of the relative ratio of the methylated allele versus the non-methylated allele. The heights of the peaks given by the pyrograms were converted into numerical values (Additional file 1: Figure S1). Methylation levels of the five CpG sites were averaged, and a single value was analyzed for each sample [23].

### Statistical analyses

Statistical analyses were performed using GraphPad Prism 5.0 (GraphPad Software Inc.). First, all groups were analyzed for normal distribution (Kolmogorov–Smirnov test), and if different sample groups followed normal distribution, *t* tests (paired and unpaired) were used; otherwise, the Wilcoxon matched-pairs test was used in the case of paired samples (WT versus kidney), and the Mann–Whitney test was used for unpaired samples. For comparisons with more than two groups, we used the Kruskal–Wallis test with Tukey’s multiple comparison post-test correction.

A receiver operating characteristic (ROC) curve was created to determine the LINE-1 methylation level threshold that could discriminate cases with relapse from those without relapse. To analyze the relation between prognostic factors as well as LINE-1 methylation levels and relapse, odds ratios (ORs) and 95% confidence intervals (CIs) were calculated by unconditional logistic regression analysis using SPSS version 21.0 (IBM). All differences were considered statistically significant if *p* < 0.05.

## Results

### Hypomethylation of LINE-1 in WTs

We analyzed 47 FFPE WTs and normal kidney paired samples. There were 31 and 16 patients classified as intermediate and high risk, respectively, among which 17 experienced relapse.

**Fig. 1:**
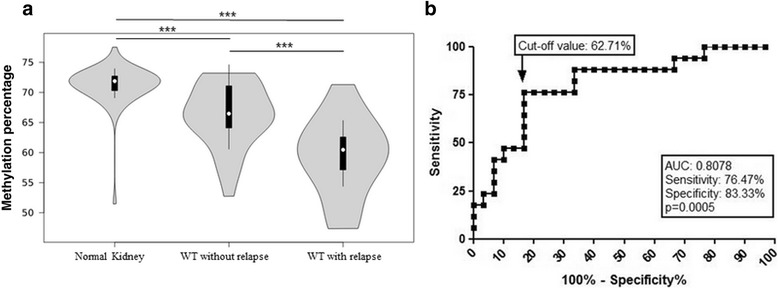
LINE-1 methylation profile in WT according to relapse status. **a** Violin plots showing LINE-1 methylation levels in normal kidney and WT samples grouped according to relapse status. The curve is estimated by a kernel density and is proportional to the number of samples. Internal boxplots include methylation levels within the 25% and 75% interquartiles, with bars indicating 1.96 × standard deviation and white dots representing the median methylation values. ***p < 0.0001, Kruskal–Wallis test. **b** ROC curve of LINE-1 methylation for discrimination of WT samples without relapse and WT samples with relapse (*p* < 0.001)

Methylation levels of five CpG sites located in the LINE-1 sequence were evaluated by pyrosequencing in matched WT blastemal component and renal cortex tissues. WT samples presented a hypomethylated pattern in all five CpG sites compared to matched kidney samples; therefore, the averaged methylation levels of the five CpG sites were used for further analyses. WT presented a hypomethylation profile (median 65.0%, 47.4–73.2%) compared to normal kidney samples (median 71.8%, 51.5–77.5%; *p* < 0.0001) (Fig. 1a).

### Association of LINE-1 hypomethylation with clinical and tumor characteristics

No significant associations were found between LINE-1 methylation levels and clinical–pathological characteristics including age at diagnosis, gender, risk classification, and tumor stage (Table 1). Also, although the presence of anaplasia was not considered for our analysis, we analyzed LINE-1 methylation levels in tumor samples with and without anaplasia. WT samples that had anaplasia (focal or diffuse) showed a lower global methylation level compared to samples without anaplasia; those presenting diffuse anaplasia had even lower LINE-1 methylation levels (Additional file 2: Table S1). No further analysis was performed regarding the presence of anaplasia because of the low number of cases (six cases).

**Table 1:** Associations between LINE-1 methylation levels and clinical–pathological characteristics

Characteristic	Group	*N* (%)	LINE-1 methylation, median in % (25th–75th percentiles)	*p* value
Gender	Male	28 (59.6)	65.11 (58.66–67.09)	0.978
	Female	19 (40.4)	64.06 (58.54–71.21)	
Age at diagnosis	0–< 2 years	14 (29.8)	66.47 (64.50–71.23)	0.258
	2–5 years	24 (51.0)	63.40 (58.32–67.17)	
	> 5 years	9 (19.2)	61.28 (56.80–67.74)	
Stage	I	11 (23.4)	62.59 (57.12–66.27)	0.281
	II	20 (42.6)	64.80 (58.45–69.87)	
	III	16 (34.0)	65.00 (63.14–71.28)	
Risk classification	Intermediate	31 (66.0)	65.23 (60.46–68.38)	0.229
	High	16 (44.0)	61.66 (57.35–67.27)	

We observed that LINE-1 methylation levels were lower in tumor samples from patients with relapse (median methylation 60.5%) compared to patients without relapse (median methylation 66.5%; *p* = 0.0005) (Fig. 1a). To explore this result further, a ROC curve analysis was applied to verify the ability of LINE-1 methylation levels to discriminate WT samples from these patients. Using a cut-off value of 62.71% for LINE-1 methylation levels, the area under the curve was 0.808, with a sensitivity of 76.5% and a specificity of 83.3% (*p* = 0.0005; Fig. 1b).

**Fig. 2:**
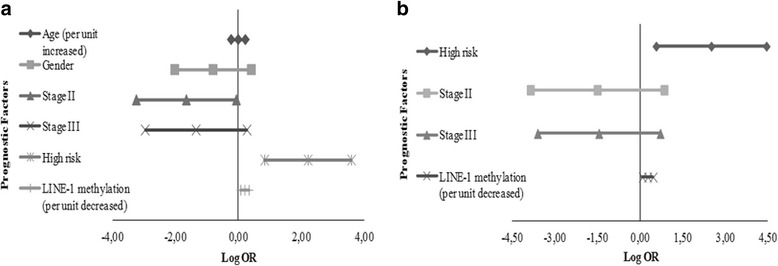
Estimated risk of relapse in WT patients according to prognostic factors and LINE-1 methylation levels. **a** Univariate risk estimates of relapse; to analyze gender as a prognostic factor, we used “male” as the reference category. **b** Adjusted risk estimates of relapse: risk classification was adjusted by stage and LINE-1 methylation levels; stage was adjusted by risk classification and LINE-1 methylation levels; and LINE-1 methylation levels were adjusted by stage and risk classification

Given that we found a difference in LINE-1 methylation levels between WT samples from patients with and without relapse in this cohort, thus we applied the uni- and multivariate analyses with other prognostic factors (stage and histological risk classification) as an exploratory question. We observed an association between high-risk classification and decreased LINE-1 methylation levels with a higher risk of relapse when we performed a univariate analysis (Fig. 2a). Hence, we applied a multivariate analysis to evaluate LINE-1 methylation levels regarding the established prognostic factors (stage and risk classification). This analysis showed that risk stratification, as expected, as well as LINE-1 methylation levels were independent variables for risk of relapse: the lower the methylation levels, the higher the risk of relapse. The logistic regression model highlighted an increase of 30% in risk of relapse per decreased unit of methylation (OR 1.30; 95% CI 1.07–1.57; Fig. 2b). In other words, a decrease of 1% in the percentage of methylated cells measured by pyrosequencing is associated with an increase of 30% in relapse risk.

## Discussion

Although WT is a curable disease, a considerable number of patients experience relapse. Thus, a refinement in the risk stratification for patients with WT could help avoiding overtreatment, improve survival chances, and reduce morbidity. Risk classification from a SIOP-2001 clinical trial is based on tumor response to chemotherapy, with blastemal component predominance classifying patients in the high-risk group [24]. However, such risk stratification has its flaws and could benefit from the addition of molecular markers of relapse. These flaws in risk stratification include central pathology review and lack of inclusion of tumor volume, with evaluation only of percent of residual cell type. Hence, a current SIOP-2001 study is evaluating if a threshold of 20–50 ml of remaining blastemal cells can be used as a new stratifying biomarker [25, 26]. However, we could not evaluate the residual blastemal volume retrospectively in this cohort.

LINE-1 serves as a surrogate marker of global methylation, and several studies have demonstrated the impact of its methylation levels on the prognosis of patients with cancer [27], such as colon [28], gastric [29], and hepatocellular [30] cancers. We observed lower LINE-1 methylation levels in tumor compared to matched normal kidney samples; however, no differences were observed when comparing the blastemal components from high- and intermediate-risk samples. Nevertheless, tumor samples from patients who experienced relapse showed lower LINE-1 methylation levels compared to tumor samples from patients without relapse. These data suggest that LINE-1 methylation levels represent a candidate biomarker of relapse for patients with WT.

Global hypomethylation has been observed in embryonal solid tumors [31, 32]. In WT, global hypomethylation in two satellite regions, satellite α and satellite 2, was reported as a surrogate marker of global methylation in all histological types [33]. Lower LINE-1 methylation levels also are associated with telomere shortening in WT compared to normal kidney samples [21]. Altogether, global DNA hypomethylation seems to be a common feature in WT [12, 21, 33], independently of the marker used or whether preoperative chemotherapy or surgery was the initial treatment. These lower methylation levels not only may be a consequence of the tumorigenesis process but also may actively contribute to tumor development and/or progression. Global DNA hypomethylation is associated with genomic instability of transposons and retrotransposons, as well as with activation of oncogenes [34, 35]. Therefore, it is not surprising that lower global methylation levels are often associated with poor prognosis in different tumor types [14] including the embryonal medulloblastomas [36] and hepatoblastomas [32, 37].

Survival rates for WT-relapsed patients may reach only 50% even under aggressive treatment [4]. Thus, factors that can predict relapse may help standardize treatment so that a more conservative approach could be applied to reduce morbidity for patients with a lower risk of relapse. For those patients with a higher relapse risk, alternative treatments must be proposed because the current therapeutic approaches reach the limits of toxicity. In patients with surgery as a first treatment, 11p15 LOI has been proposed as a biomarker for relapse in patients with stage I favorable histology WT [38, 39], while loss of heterozygosity on 11p15 was associated with a risk of recurrence of 5.00 [9]. Furthermore, a study evaluating the prognostic significance of different molecular markers (including 1q gain, 1p and 16q losses, and *MYCN* gain) in a large case series (586) of WT showed that, besides tumor stage and high-risk histology, only 1q gain is an independent predictor of event-free survival [7]. Our data showed that LINE-1 methylation levels could distinguish tumor samples from patients with and without relapse with a sensitivity of 76.5% and a specificity of 83.3%, remaining as an independent prognostic factor for relapse on top of the histological risk classification. In this cohort, the high-risk patients were classified solely based on the remaining proportion of the blastemal component (after excluding necrosis). This enrichment for blastemal cells, despite the histological risk classification, allows for comparison of all tumors with a lower influence of the cellular composition given by the different cells presented in the tumors. Therefore, it is tempting to propose that LINE-1 methylation could be a useful predictor of relapse, in addition to risk classification, helping to guide new treatment approaches. Finally, the turnover time of the analysis of approximately 10 days would enable the indication of patient’s prognosis before postoperative treatment.

Our results reinforce previous data showing a global hypomethylation profile in WT. Also, we suggest a prospective evaluation to access the feasibility of the use of LINE-1 methylation levels as a possible marker of relapse after chemotherapy treatment. Although our findings are quite promising, the number of samples was limited, and the analyses were restricted to the blastemal cells of the tumors. Thus, other studies should be carried out to confirm the prognostic value of LINE-1 methylation in WT.

## Conclusions

Our results showed that the blastemal component of WT samples exhibits a LINE-1 hypomethylation pattern in comparison to normal kidney samples. In addition, these lower methylation levels not only may be a consequence of the tumorigenesis process but also may actively contribute to tumor development and/or progression. This association suggests that embryonal tumors are driven by different oncogenic mechanisms, as has been observed in other embryonal tumors. We also show that with each unit decrease in global methylation, the chances of relapse increase, indicating the accuracy of this marker as a possible relapse predictor in association with the currently used risk classification (SIOP 2001). However, because of the small number of samples and the fact that these analyses were done only in the blastemal component, more studies need to evaluate prospectively the efficacy of this molecular marker.

## Additional file

Additional file 1: Figure S1. LINE-1 methylation pyrograms of representative samples. (A) Normal kidney; (B) Wilms tumor. Five CpG sites were evaluated in the LINE-1 promoter sequence. Arrows indicate internal controls for bisulfite conversion. (TIFF 416 kb)
Additional file 2: Table S1. Updated clinical data and methylation mean of the 5 LINE-1 sites of each patient. (DOCX 33 kb)
