# Supplementary figures and images for: Association between long interspersed nuclear element-1 methylation levels and relapse in Wilms tumors
Source: Clin Epigenetics. 2017 Dec 12;9:128. doi: 10.1186/s13148-017-0431-6 (PMC5728012; doi:10.1186/s13148-017-0431-6)

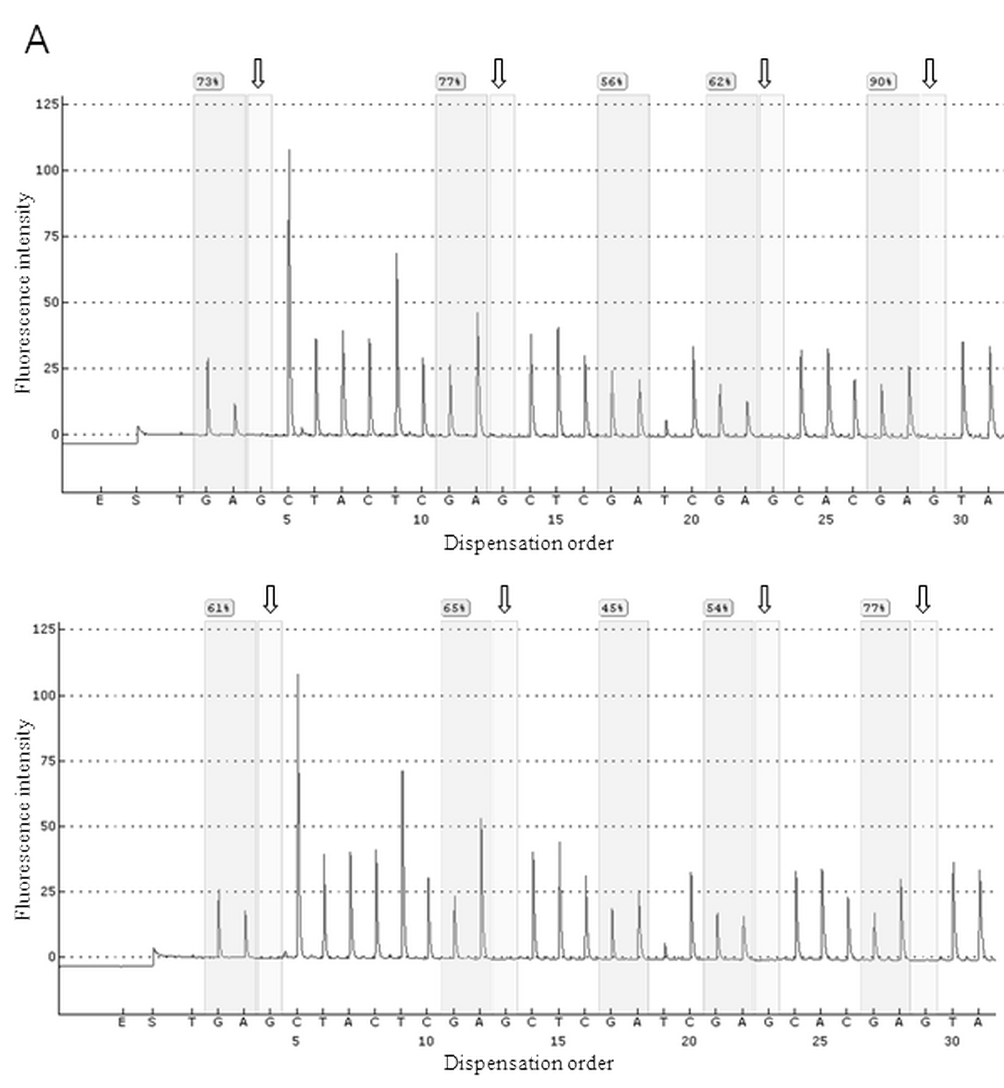

Supplement: Additional file 1: Figure S1. — LINE-1 methylation pyrograms of representative samples. (A) Normal kidney; (B) Wilms tumor. Five CpG sites were evaluated in the LINE-1 promoter sequence. Arrows indicate internal controls for bisulfite conversion. (TIFF 416 kb) [file 13148_2017_431_MOESM1_ESM.tif]
