# Supplementary material for: Association between long interspersed nuclear element-1 methylation levels and relapse in Wilms tumors
Source: Clin Epigenetics. 2017 Dec 12;9:128. doi: 10.1186/s13148-017-0431-6 (PMC5728012; doi:10.1186/s13148-017-0431-6)
Supplement: Additional file 2: Table S1. — Updated clinical data and methylation mean of the 5 LINE-1 sites of each patient. (DOCX 33 kb) [file 13148_2017_431_MOESM2_ESM.docx]

| ID Pacient | Age at diagnosis  (years) | Gender | Stage | Risk Classification | Anaplasia | Relapse | L1 methylation Tumor samples (Mean) | Status |
| --- | --- | --- | --- | --- | --- | --- | --- | --- |
| 01 | 13 | M | II | High | No | No | 67.1 | Alive |
| 02 | 4 | F | III | High | Yes (Diffuse) | Yes | 48.4 | Dead |
| 03 | 4 | F | I | Intermediate | No | No | 65.7 | Alive |
| 04 | 4 | M | II | Intermediate | No | No | 58.3 | Alive |
| 05 | 3 | F | III | Intermediate | No | Yes | 65.0 | Alive |
| 06 | 7 | M | II | Intermediate | No | Yes | 47.4 | Alive |
| 07 | 3 | M | II | Intermediate | No | No | 66.2 | Alive |
| 08 | 4 | M | I | High | No | Yes | 62.6 | Alive |
| 09 | 5 | M | II | Intermediate | No | No | 71.7 | Alive |
| 10 | 1 | F  **Supplemental Table 1.** Updated clinical data and methylation mean of the 5 LINE-1 sites of each patient | III | Intermediate | No | No | 64.1 | Alive |
| 11 | 2 | M | III | Intermediate | No | No | 66.7 | Alive |
| 12 | 2 | M | III | Intermediate | No | Yes | 64.9 | Alive |
| 13 | 1 | F | III | Intermediate | No | No | 72.9 | Alive |
| 14 | 4 | M | II | Intermediate | No | No | 56.5 | Alive |
| 15 | 3 | F | II | Intermediate | No | Yes | 58.4 | Dead |
| 16 | 5 | M | I | High | No | Yes | 58.0 | Alive |
| 17 | 5 | M | III | Intermediate | Yes (Focal) | No | 62.8 | Alive |
| 18 | 3 | M | II | Intermediate | No | No | 66.7 | Alive |
| 19 | 12 | M | III | Intermediate | No | No | 73.2 | Alive |
| 20 | 4 | F | II | Intermediate | No | No | 63.9 | Alive |
| 21 | 3 | M | I | High | Yes (Focal) | Yes | 57.1 | Alive |
| 22 | 11 | M | III | Intermediate | No | No | 59.8 | Alive |
| 23 | 2 | M | III | Intermediate | No | No | 64.6 | Alive |
| 24 | 4 | F | II | Intermediate | No | No | 65.6 | Alive |
| 25 | 2 | M | II | High | No | No | 67.0 | Alive |
| 26 | 2 | F | III | High | Yes (Focal) | Yes | 58.1 | Dead |
| 27 | 6 | F | II | High | No | Yes | 60.6 | Dead |
| 28 | 2 | F | II | Intermediate | No | No | 71.7 | Alive |
| 29 | 4 | F | II | Intermediate | Yes (Focal) | No | 58.5 | Alive |
| 30 | 4 | M | II | Intermediate | No | No | 72.5 | Alive |
| 31 | 4 | M | II | Intermediate | No | No | 70.8 | *Lost of follow up |
| 32 | 5 | F | II | High | No | Yes | 62.0 | Lost of follow up |
| 33 | 2 | M | I | Intermediate | No | No | 66.3 | Alive |
| 34 | 1 | F | III | Intermediate | No | Yes | 71.3 | Dead |
| 35 | 3 | F | II | Intermediate | No | Yes | 60.7 | Alive |
| 36 | 9 | M | I | High | No | Yes | 61.3 | Alive |
| 37 | 6 | F | III | Intermediate | No | No | 68.4 | Alive |
| 38 | 6 | M | I | High | No | Yes | 53.8 | Lost of follow up |
| 39 | 7 | M | III | Intermediate | No | No | 64.2 | Alive |
| 40 | 2 | M | I | Intermediate | No | No | 67.0 | Alive |
| 41 | 4 | F | I | High | Yes (Diffuse) | Yes | 50.2 | Alive |
| 42 | 4 | M | I | High | No | Yes | 67.3 | Alive |
| 43 | 5 | M | III | High | No | No | 71.6 | Alive |
| 44 | 2 | M | I | Intermediate | No | No | 65.2 | Alive |
| 45 | 2 | F | III | High | No | No | 71.2 | Alive |
| 46 | 5 | F | II | High | No | No | 72.6 | Alive |
| 47 | 2 | M | II | Intermediate | No | No | 52.7 | Alive |

*The only case with loss of follow up that did not present relapse, had a follow-up of 10 years until it was lost.
